# Roles of Ambient Temperature and PM_2.5_ on Childhood Acute Bronchitis and Bronchiolitis from Viral Infection

**DOI:** 10.3390/v14091932

**Published:** 2022-08-30

**Authors:** Pei-Chun Chen, Chih-Hsin Mou, Chao W. Chen, Dennis P. H. Hsieh, Shan P. Tsai, Chang-Ching Wei, Fung-Chang Sung

**Affiliations:** 1 Department of Public Health, China Medical University College of Public Health, Taichung 406, Taiwan; 2 Management Office for Health Data, China Medical University Hospital, Taichung 404, Taiwan; 3 University of Maryland Global Campus, Adelphi, MD 20783, USA; 4 Department of Environmental Toxicology, University of California, Davis, CA 95616, USA; 5 School of Public Health, Texas A&M University, College Station, TX 77843, USA; 6 Department of Pediatrics, China Medical University College of Medicine, and Department of Pediatrics, Children’s Hospital of China Medical University Hospital, Taichung 404, Taiwan; 7 Department of Health Services Administration, China Medical University College of Public Health, Taichung 406, Taiwan; 8 Department of Food Nutrition and Health Biotechnology, Asia University, Taichung 413, Taiwan

**Keywords:** acute bronchitis and bronchiolitis, ambient temperature, fine particulate matter, human respiratory syncytial virus, temperature inversion

## Abstract

Studies have associated the human respiratory syncytial virus which causes seasonal childhood acute bronchitis and bronchiolitis (CABs) with climate change and air pollution. We investigated this association using the insurance claims data of 3,965,560 children aged ≤ 12 years from Taiwan from 2006–2016. The monthly average incident CABs increased with increasing PM_2.5_ levels and exhibited an inverse association with temperature. The incidence was 1.6-fold greater in January than in July (13.7/100 versus 8.81/100), declined during winter breaks (February) and summer breaks (June–August). The highest incidence was 698 cases/day at <20 °C with PM_2.5_ > 37.0 μg/m^3^, with an adjusted relative risk (aRR) of 1.01 (95% confidence interval [CI] = 0.97–1.04) compared to 568 cases/day at <20 °C with PM_2.5_ < 15.0 μg/m^3^ (reference). The incidence at ≥30 °C decreased to 536 cases/day (aRR = 0.95, 95% CI = 0.85–1.06) with PM_2.5_ > 37.0 μg/m^3^ and decreased further to 392 cases/day (aRR = 0.61, 95% CI = 0.58–0.65) when PM_2.5_ was <15.0 μg/m^3^. In conclusion, CABs infections in children were associated with lowered ambient temperatures and elevated PM_2.5_ concentrations, and the high PM_2.5_ levels coincided with low temperature levels. The role of temperature should be considered in the studies of association between PM_2.5_ and CABs.

## 1. Introduction

Childhood acute bronchitis and bronchiolitis (CABs) cause inflammation and swelling of the bronchi and bronchioles, respectively, occurring mainly due to viral infections attributable to the respiratory syncytial virus (RSV), rhinovirus, influenza infection, etc. [1,2,3,4,5,6]. Children are more vulnerable to the infection and may suffer from subsequent complications [5,6]. The events of CABs are presented with seasonality, associated with the change in ambient meteorological conditions. It generally occurs in colder months in the UK, the US, Japan, Spain, etc. [1,4,7,8]. A study in Spain showed the risk of RSV in 1495 infants peaked at a mean temperature of 9 °C or lower [8]. A Chinese study in Suzhou found a strong inverse relationship between RSV infection in hospitalized children and monthly average temperatures (r = −0.84) [9]. A systematic review and meta-analysis showed that RSV infection was greater in winter and spring among 489,641 children with acute respiratory tract infections in China [10]. However, the infection in some parts of tropical areas may peak in the rainy season [11].

Air pollution exposure is a critical public health concern associated with harmful respiratory disorders [12,13,14,15,16,17]. Among ambient air pollutants, particulate matter (PM) with an aerodynamic diameter of ≤2.5 μm (PM_2.5_) and 10 μm (PM_10_) and other pollutants have attracted attention for its association with childhood respiratory health. A US study in Atlanta evaluated 503,004 children aged 0–4 years old with emergency visits and found that traffic pollutants, the carbon component of PM_2.5_ and ozone, might exacerbate bronchitis and bronchiolitis, pneumonia, and upper respiratory infections [12]. An earlier Czech study found that polycyclic aromatic hydrocarbons and PM_2.5_ were associated with an increase in bronchitis risk for children less than 2 years old, with the risk being greater in colder seasons than in summer [13]. A study using hospital data from Hefei, China, found the risk of clinic visits for childhood acute bronchitis was significantly associated with traffic-related NO_2_, PM_2.5_, and CO in the cold season [15]. The study in subtropical Hong Kong found hospitalizations for acute bronchiolitis in children were associated with temperature and exposure to NO_2_ and PM_10_ [16]. A Brisbane study using time-stratified case-crossover analysis revealed that the air pollution associated risk of acute upper respiratory infections in children increased during the cold season and at night, whereas the risk of acute lower respiratory infections appeared during the daytime in the warm season [17]. 

The atmospheric dispersion of air pollutants is associated with temperature, wind speed, humidity, and precipitation [18,19,20]. The compositions and concentrations of PMs exhibit remarkable seasonal variations, higher in cold months than in hot months. 

The inverse relationship between ambient temperature and air quality has been considered in studies evaluating the impact of these two factors on health outcomes for adults, including cardiovascular and respiratory disorders [21,22,23,24,25]. Studies on seasonal childhood acute respiratory infection highlighted air-pollutant-induced oxidative stress and inflammation as the mechanism which enhances acute respiratory infection from virus or bacteria [12,15,16,17]. Studies have investigated the association between the spread of the pathogen among children and their activity and behavior, which might be mediated by ambient conditions and pollutants [26]. Therefore, we aimed to assess the risk of CABs, which is usually caused by viral infection, in association with both ambient temperature and PM_2.5_ in children < 12 years old.

## 2. Methods and Materials 

### 2.1. Data Source and Study Population 

This study used longitudinal claims data from the National Health Insurance program which is a mandatory program for all residents of Taiwan. Data were obtained from the Health and Welfare Data Science Center, comprising health care records of the insured population from 1996 to 2016. Demographic data regarding age, sex, residential area, parental income, and medical records of disease diagnosis, treatment, medication, and costs of inpatient and outpatient care were available. Diseases were recorded using the International Classification of Diseases, 9th/10th Revisions, Clinical Modification (ICD-9-CM/ICD-10-CM). From medical records of the insurance claims data from 2006 to 2016, children diagnosed with acute bronchitis (ICD-9CM 466.0 and ICD-10-CM J20.8 and J20.9) and acute bronchiolitis (ICD-9-CM 466.11 and 466.19, and ICD-10-CM J21.0, J21.1, J21.8 and J21.9) were identified. These patients were analyzed to identify relationships between CABs and ambient environmental conditions.

All personal information in the data files was anonymized, and surrogate identification numbers were used to ensure patients’ privacy. The Research Ethics Committee of China Medical University and Hospital approved the study (H107257). Because personal identification information was removed from the claims data, the requirement for patient consent was waived. This study was carried out in accordance with the Declaration of Helsinki.

### 2.2. Environmental Condition Data

The Taiwan Environmental Protection Administration (EPA) has established air quality monitoring stations at 76 locations in 20 administrative regions since 1993 to measure hourly ambient air pollutants. Meteorological conditions, such as temperature and humidity, were also recorded. However, hourly PM_2.5_ was not routinely monitored until 2006. Therefore, we obtained from the EPA web, the records of hourly measurements of daily ambient PM_2.5_, sulfur dioxide (SO_2_), and ozone (O_3_) levels, for the period 2006–2016, for this study. 

Most of the monitoring stations were established in residential areas in cities and townships to measure the hourly air quality. Monitoring systems were also established to measure emissions from road traffic and industry activities. The number of monitoring stations ranged from 3 to 11 based on the urbanization of the city and county; one monitoring station was established in each of the two rural counties along the mountainous east coast of Taiwan. Detailed information on monitoring instruments, stations, and quality control and assurance is available on the Taiwan EPA website (https://taqm.epa.gov.tw/taqm/en/default.aspx, accessed on 25 November 2018).

### 2.3. Statistical Analysis

From the claims data, we identified 3,965,560 children aged ≤ 12 years from 2006–2016 for this study. Children with missing data on sex and birthdate or from areas without complete data of ambient temperatures were excluded from the data analyses. Other diseases of the respiratory system not classified elsewhere (ICD-9-CM 519.8) were excluded. Data analysis first involved calculating and plotting the monthly incidence of CABs per 100 children from January to December annually for the period from 2006–2016. The seasonal cyclic patterns of CABs infection were similar yearly. Thus, we pooled the 11-year data to calculate and plot the mean monthly incident CABs by the monthly mean temperature and PM_2.5_ level. The hourly values of temperature and PM_2.5_ available from all stations were pooled to calculate the monthly means to represent the conditions general population might have been expose to. The age- (≤2, 3–5, and 6–12 years) and sex-specific incidence of CABs cases per day were estimated by temperature stratum (<20, 20–24, 25–29, and ≥30 °C, representing thermally cold, comfortable, warm, and hot, respectively). Days with these temperature levels were also specified. Overall average daily cases in 11 years were estimated by temperature levels, quartile PM_2.5_ levels (<15.0, 15.0–23.6, 23.7–36.9, and ≥37.0 μg/m^3^), parental incomes for insurance premiums (<250,000, 250,000–299,999, and >300,000 NTD), SO_2_, and O_3_. Poisson regression analysis was used to estimate crude relative risk (cRR) and 95% confidence interval (CI) of CABs associated with these variables [27].

**Figure 1:**
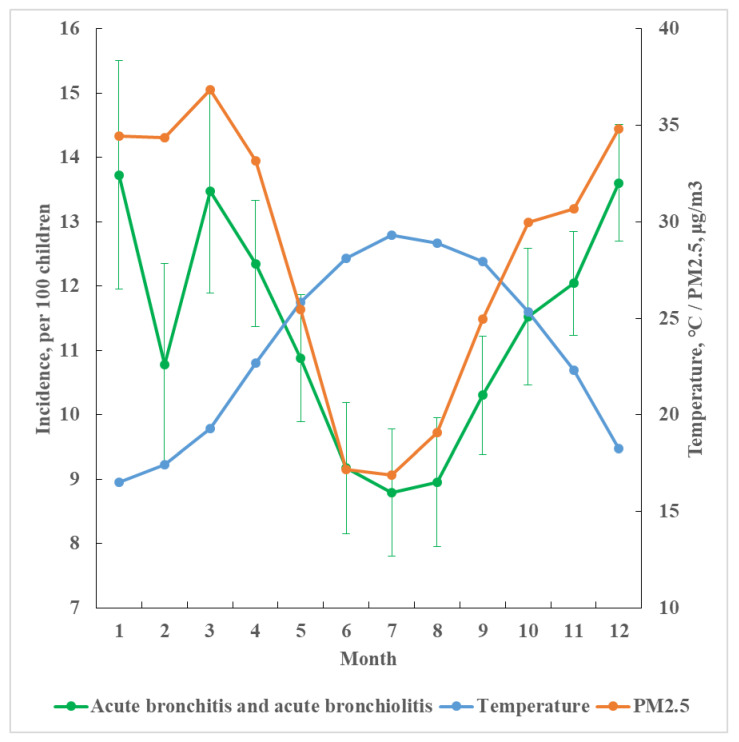
Monthly average incidence rate with standard deviation of childhood acute bronchitis and bronchiolitis by monthly average temperature and PM_2.5_ level from 2006–2016 in Taiwan.

**Figure 2:**
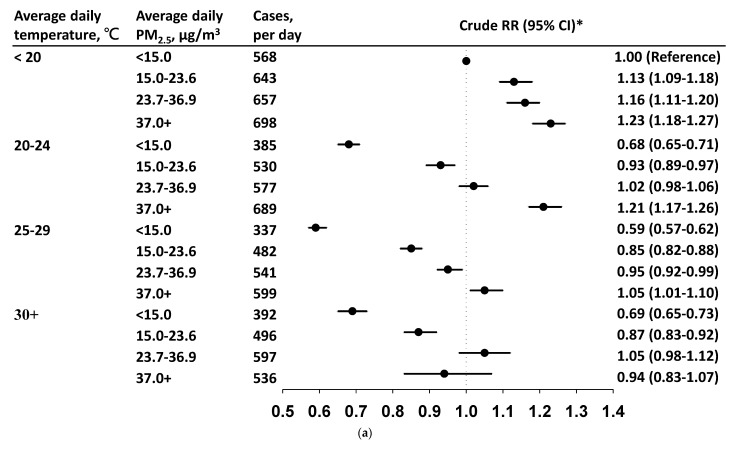

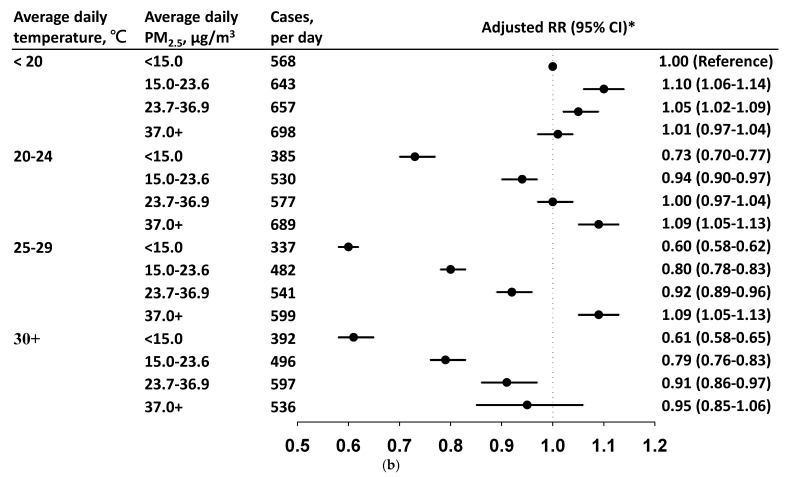
(**a**) Average daily incidence and crude relative risk (RR) and 95% confidence interval (CI) of acute bronchitis and bronchiolitis by PM_2.5_ level in each temperature stratum. (**b**) Average daily incidence of acute bronchitis and acute bronchiolitis by PM_2.5_ level in each temperature stratum and adjusted relative risk (RR) and 95% confidence interval (CI) after controlling for income, SO_2_ level, and O_3_ level.

**Figure 3:**
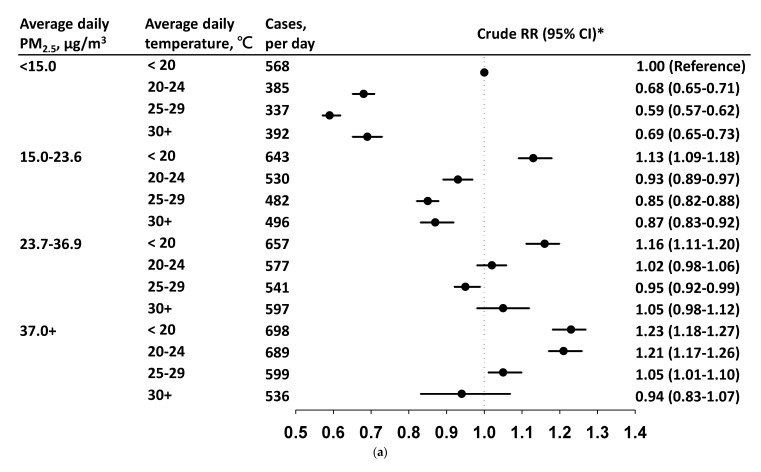

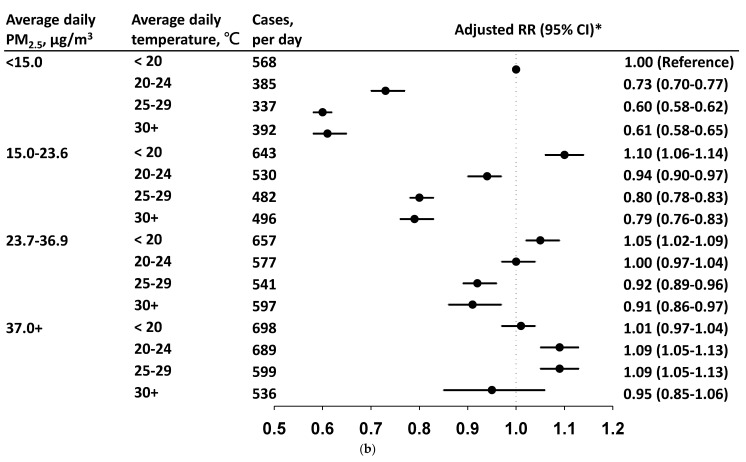
(**a**) Average daily incidence and crude relative risk (RR) and 95% confidence interval (CI) of acute bronchitis and bronchiolitis by temperature level in each PM_2.5_ stratum comparing the reference condition with PM_2.5_ <15.0 μg/m^3^ at temperatures <20 °C. (**b**) Average daily incidence and adjusted relative risk (RR) and 95% confidence interval (CI) of acute bronchitis and bronchiolitis by temperature level in each PM_2.5_ stratum comparing the reference condition with PM_2.5_ <15.0 μg/m^3^ at temperatures <20 °C after controlling for income, SO_2_ level, and O_3_ level.

We further estimated the daily incidence cases of CABs by the PM_2.5_ stratum in each temperature stratum. The cRR of CABs at each specific temperature/PM_2.5_ stratum was calculated using the CABs incidence at the stratum with <20 °C and PM_2.5_ < 15.0 μg/m^3^ as the reference condition. The adjusted relative risk (aRR) of CABs at each specific stratum was calculated after controlling for sex, age, income, SO_2_ level, and O_3_ level. We also estimated the incidence cases and RRs of CABs by temperature stratum in each PM_2.5_ stratum, and the stratum with <20 °C and PM_2.5_ < 15.0 μg/m^3^ was also used as the reference condition. The estimated incidence cases and cRRs and aRRs of CABs were also plotted. Moreover, the lag effects of temperature and PM_2.5_ changes on CAB risks for 1, 3, and 7 days were evaluated, and the results were plotted in Supplementary Figure S1. We used SAS version 9.4 (SAS Institute, Inc., Cary, NC, USA) to perform data analysis; two-tailed *p* < 0.05 was considered statistically significant. The Poisson regression analysis used proc genmod to measure RRs. We used Excel to plot Figure 1 and used Systat (Systat Software, Inc., San Jose, CA, USA) to plot Figure 2a,b and Figure 3a,b, and the Supplementary Figure S1.

## 3. Results

### 3.1. Monthly Incidence by Temperature and PM_2.5_ Levels and Daily Cases by Age, Temperature, and Sex 

The monthly average temperature ranged from 17.5 °C in January to 29.2 °C in July during the period from 2006 to 2016 (Figure 1). The monthly average incidence rates of CABs over 11 years were inversely associated with the average monthly temperature, 1.6-times higher in January than in July (13.7 vs. 8.81 per 100, *p* < 0.01). The monthly incidence rates of CABs were almost parallel to PM_2.5_ levels, except in February, when the incidence dropped to 10.8 per 100 (*p* < 0.01 compared with 13.7).

During the period from 2006 to 2016, the overall daily CABs incidence was higher in boys than in girls (1190 versus 988 per day, *p* < 0.001) (Table 1). The age- and sex-specific daily incidence of CABs cases declined with the increase in temperature, with the lowest cases when it was 25–29 °C. For all children, the daily incidence of CABs cases declined from 355 to 257 per day in boys (*p* < 0.001) and from 297 to 212 per day in girls (*p* < 0.001) when the temperature increased from <20 °C to 25–29 °C.

**Table 1:** Average daily cases of acute bronchitis and bronchiolitis by age, temperature, and sex from 2006–2016 in Taiwan.

			Bronchitis			Bronchiolitis			Both		
			Boy	Girl	*p*	Boy	Girl	*p*	Boy	Girl	*p*
Age	Temperature	Days	n/Day	n/Day		n/Day	n/Day		n/Day	n/Day	
<2	<20 °C	1017	46	36	0.0006	25	19	0.0002	71	55	0.0004
	20–24	1036	47	37		23	18		70	55	
	25–29	1654	41	32		20	15		61	47	
	30+	249	43	34		22	16		65	50	
3–5	<20 °C	1017	104	88	0.0009	17	15	0.0002	121	103	0.0008
	20–24	1036	96	82		15	13		111	95	
	25–29	1654	82	69		13	11		95	80	
	30+	249	84	71		13	11		97	82	
6–12	<20 °C	1017	145	124	0.0002	17	15	0.0004	162	139	0.0002
	20–24	1036	120	101		14	12		134	113	
	25–29	1654	90	75		11	9		101	84	
	30+	249	89	74		11	9		100	83	
All	<20 °C	1017	295	249	0.0002	60	48	0.0003	355	297	0.0002
	20–24	1036	263	220		53	43		316	263	
	25–29	1654	213	177		44	35		257	212	
	30+	249	216	179		46	37		261	216	
	Total	3956 *	987	825		203	163		1190	988	

*p* value refers to significance level of coefficient of relationship of daily incidence cases between boys and girls associated with temperatures by age group. * Information on temperature was not fully available for 62 days from 2006 to 2016.

### 3.2. Temperature, PM_2.5_, and Income Specific Daily Incident Cases

Table 2 shows the overall average daily incidence and relative risk of CABs by temperature, PM_2.5_, parental income, five ppb increment of SO_2_, and five ppb increment of O_3_. The average daily cases of CABs decreased with increasing temperature, from 652 cases/day at <20 °C to 477 cases/day at ≥30 °C with a cRR of 0.73 (95% CI = 0.71–0.76)]. In contrast, the average cases of CABs increased with increasing level of PM_2.5_, from 393 cases/day at <15 μg/m^3^ to 666 cases/day at 37+ μg/m^3^ with a cRR of 1.69 (95% CI = 1.66–1.73). The incidence rate of CABs increased with parental income and SO_2_, but not O_3_.

### 3.3. Daily Incidence and Risk by PM_2.5_ Level in Each Temperature Stratum

Figure 2a,b show the daily cases, and cRRs and aRRs, respectively, of CABs by the daily PM_2.5_ level within each temperature stratum. In each temperature stratum, the daily CABs incidence increased with PM_2.5_. The CABs incidence was 568 cases/day when ambient temperature was <20 °C with PM_2.5_ < 15.0 μg/m^3^ (reference condition). In this temperature stratum, the cRRs were in a rising trend by the PM_2.5_ level (Figure 2a). However, the linear relationship between PM_2.5_ and CABs disappeared after controlling for income, SO_2_, and O_3_ level_._ The risk decreased to 1.01 (95% CI = 0.97–1.04) at PM_2.5_ levels of 37.0+ μg/m^3^. At temperatures of 20–24 °C and 25–29 °C, the increased risk of CABs was only noted when PM_2.5_ was ≥ 37 μg/m^3^ after controlling for income, SO_2_ level, and O_3_ level. Adjusted RRs were all <1.00 at a temperature of 30 °C, although increased with PM_2.5_ level: from 0.61 (95% CI = 0.58–0.65) for a PM_2.5_ level of <15 μg/m^3^ to 0.95 (95% CI = 0.85–1.06) when PM_2.5_ was ≥ 37.0 μg/m^3^. Figure 3a,b show that aRRs of CABs were significant only at temperatures of <20 °C when PM_2.5_ levels were < 37.0 μg/m^3^, or at temperatures of 20 °C–29 °C when PM_2.5_ levels were ≥37.0 μg/m^3^, with temperatures of <20 °C and PM_2.5_ levels of <15 μg/m^3^ as the reference condition.

The lag effect data revealed that the RRs of CABs associated with temperature and PM_2.5_ were lower on lag-3 day than on lag-1 day and lag-7 day when temperatures were lower than 30 °C after income, SO_2_ level, and O_3_ level were controlled for (Supplementary Figure S1).

Supplementary Figure S1 Lag 1-, 3- and 7-day adjusted relative risk (RR) and 95% confidence interval (CI) of acute bronchitis and acute bronchiolitis associated with temperature and PM_2.5_ level controlling for income, SO_2_ level, and O_3_ level. 

## 4. Discussion

The ambient PM_2.5_ concentrations had a strong inverse correlation with ambient temperatures and were higher in cold months than in warmer months in Taiwan [18]. Our data revealed that CABs infections were statistically associated with these two factors. The incidence was higher in colder months when the PM_2.5_ levels were higher, and the incidence was lower in the hot months when the PM_2.5_ levels were lower. The CABs incidence showed a stronger relationship with temperature than PM_2.5_ after adjusting for potential confounders.

In this study, the highest CABs incidence was noted when the daily average temperature was <20 °C, with a high ambient PM_2.5_ concentration. With a subtropical climate, ambient air temperatures <15 °C account for the lowest 5% of the temperature distribution and are considered cold extremes in Taiwan [24,25]. In cold months, cold air at the Earth’s surface is often inversely overlain by warm air, particularly when the weather is not sunny or windy. The probability of inversion may exceed 70% in the cold months in most areas in Taiwan [28]. The probability of inversion decreases to nearly 20% in hot months with inversion occurring only in the morning. In general, when a temperature inversion occurs, PM_2.5_ exhibits constrained dispersion and accumulates near the ground [29]. 

Our study showed that the CABs incidence increased with the ambient PM_2.5_ concentration regardless of the temperature range. For the temperature of <20 °C, the daily increase in CABs cases by the PM_2.5_ level, but the corresponding aRRs appeared to have an inverse U-shaped association. The lowest CABs incidence cases appeared when the daily values of temperature ranged from 25 °C to 29 °C, which could be regarded as a warm temperature range. A protective relationship was noted, when temperature ranged between 25 °C and 29 °C or over 30 °C, even at a PM_2.5_ level of ≥37 μg/m^3^ compared with temperatures of <20 °C and PM_2.5_ levels of <15 μg/m^3^. We suspect that children are more likely indoors on days with higher PM_2.5_ levels so the person-to-person infection increases. The CABs incidence may seem to have a stronger relationship with temperature than with PM_2.5_ level. Therefore, temperature should be considered during the assessment of the effects of PM_2.5_ on health. 

The mechanisms underlying the association of CABs with both PM_2.5_ level and temperature have not been well investigated [12]. 

To the best of our knowledge, no study has reported that bronchitis and bronchiolitis pathogens, that is, the human RSV, can be an airborne virus carried by ambient PMs. On the other hand, Sato et al. conducted a survey among children with acute respiratory symptoms at a pediatric outpatient clinic in Japan and found 37.1% of 499 nasopharyngeal aspirate samples were human RSV positive, which peaked in three winter seasons [7]. A US study at a childcare center found that respiratory virus was detected in 82% of 523 symptomatic episodes of serious respiratory infections and 70% of 127 asymptomatic children [26]. Half of the children had an RSV infection within 6 days after the first case was diagnosed, indicating a rapid viral spread among children, which is not likely to be spread through ambient PMs.

Therefore, the most commonly identified contagious virus associated with CABs could be RSV, which can easily and rapidly spread through direct person-to-person contacts [7]. Human-associated viruses are dominant in the indoor environment in cold months [26,30,31]. A US study found the presence of airborne RSV in daycare centers in winter [30], matching the seasonal CABs epidemic in the present study. Our data revealed that 83.2% of patients with CABs were diagnosed with acute bronchitis. We found that 56.5% of CABs cases were diagnosed when it was colder, mainly in November, December, January, February, and March, which are the cold months on the subtropical island of Taiwan. A recent study investigating the etiology of viral infections at a medical center in northern Taiwan found that RSV was the most prevalent agent among all positive viral samples (31.7% or 113/357), mainly in children <5 years old [32]. 

Our data revealed that the bronchitis incidence rate increased with age, whereas the bronchiolitis incidence rate decreased with age. These children are most likely attending daycare centers, preschools, or elementary schools. During winter or on foggy days, children are more likely to spend more time indoors than outdoors, increasing person-to-person contacts. Given that viruses causing CABs are contagious, viral infections may spread among children in daycare centers and schools on cold days.

Previous studies have reported that human occupancy is the main driver of airborne RSV in daycare centers and is dominant in winter [30,31]. It is likely that CABs are mainly caused by viruses spread through direct child-to-child contacts or indoor bioaerosols, when in near proximity to a person. 

It is also worth noting that the median national RSV infection in the US peaks in February [4]. February is a cold month in North America, and children attend daycare centers and schools during this time. Cold temperatures promote indoor activities and frequent interpersonal contacts, and thereby increase pathogen spread. By contrast, our data revealed that the average incidence of CABs exhibits an apparent dip in February, more than 20% lower than that in January and March. This is likely due to Chinese New Year celebrations, which usually occur in February, when daycare centers and schools in Taiwan are closed for the winter break; the spread of infections is thus reduced among children. The incidence climbed to a peak in March because indoor gatherings increased again among children in classes of the new school semester. The incidence was the lowest in July and August, the hot months in Taiwan, with school closure for the summer break. Thus, the spread of viral infections is lower among children in February than in March, and the lowest in July and August. 

Studies in Czech, Hefei, Hong Kong, and Brisbane suspected that air pollutants could induce oxidative stress, inflammation, and lesions in the respiratory tract, leading to pathogen-induced infection, such as by RSV, adenovirus, and/or bacteria [13,15,16,17]. The Hong Kong study reported high temperature and high NO_2_ level as risk factors of acute bronchiolitis-related hospitalization in children [16]. However, this study also reported that acute bronchiolitis admissions peaked in colder months, sharing a pattern similar to the pattern in Taiwan. Both Hong Kong and Taiwan are islands located on the western Pacific Ocean characterized by a subtropical climate. The seasonality of respiratory infection in temperate regions is because children are more likely to have indoor gatherings in colder months, increasing the pathogen spread. The Brisbane study used time-stratified case-crossover analysis to comprehensively examine risks of nine types of respiratory infections associated with hourly concentrations of four air pollutants [17]. Multiple testing might have caused a problem in this study by simultaneously using a set of multiple statistical inferences. Thus, the study found a moderate significant risk of acute bronchitis in the warm season associated with daytime NO_2_ pollution exposure for 13–24 h among children in Brisbane.

It is also interesting to note that in our study CABs infection increased with parental income. In Taiwan, after-school care or tutoring programs are commonly available as private institutes for children from kindergarten through to sixth-graders after their school-day. An earlier study found fees and locations were factors of concern from the perspective of parents [33]. We suspect that children from higher income families might be more likely than those from lower income families to attend after-school programs, leading to more frequent indoor gatherings and exposure to pathogens.

### Strength and Limitations

The strength of this study is the use of a real-world population’s claims database to provide real-world evidence of CABs risk related to ambient conditions. The stratified analysis enabled us to evaluate CABs risk by PM_2.5_ stratum in each temperature stratum, or by temperature stratum in each PM_2.5_ stratum.

However, there were limitations in this study. First, information on the daily activities of individual children and laboratory data are unavailable in the claims database, precluding in-depth analysis of interpersonal contact among children on its relationship with CABs incidence. We were unable to determine the pathogens associated with CABs without the laboratory data. The illness of bronchiolitis is mainly associated with RSV infection in children aged 5 years and younger [31], whereas the illness of bronchitis is associated RSV and other pathogens [1,2,3,4,5,6]. This study was unable to evaluate in detail the variation of CABs risks by pathogen among age groups. Second, we pooled all data available from 76 monitoring stations to measure the overall PM_2.5_ levels and temperature for the entire Taiwan area. The climate is slightly warmer in southern Taiwan than in northern areas. We did not evaluate the variation of CABs risks among areas. Third, there are other types of particle matter and pollutants which present health concerns. Other pollutants and PM_2.5_ may be collinear. Findings in Figure 2 showed incidence cases of CABs increased with increasing PM_2.5_ when temperatures were <20 °C. The corresponding aRR were opposite, and reduced after controlling for family income, SO_2_ level, and O_3_ level. The reason for this reversed pattern is not immediately clear based on the current data. However, our study findings may not be generalizable to other regions or countries. CABs infection associated with the environment factors shown in our study deserves further exploration in other regions. 

## 5. Conclusions

In this population-based study in Taiwan, seasonal CABs infections in children were associated with lower ambient temperatures, which coincide with elevated PM_2.5_ concentrations. The association of CABs may be stronger with temperature than with PM_2.5_ levels. This study suggested that the role of temperature and its related changes in human daily activities and behavior should be considered in studies of associations between PM_2.5_ and CABs.

## Figures and Tables

**Table 2:** Average daily cases and crude relative risk of acute bronchitis and acute bronchiolitis, estimated by temperature, PM_2.5_, income, and SO_2_ and O_3_ increment from 2006–2016 in Taiwan.

Variable	Rate, n/Day	Crude RR(95% CI)
Average daily temperature, °C		
<20	652	1.00 (Reference)
20–24	578	0.89 (0.87–0.90)
25–29	469	0.72 (0.71–0.73)
30+	477	0.73 (0.71–0.76)
Average daily PM_2.5_, μg/m^3^		
<15	393	1.00 (Reference)
15–23.6	528	1.34 (1.32–1.37)
23.7–36.9	587	1.49 (1.46–1.52)
37+	666	1.69 (1.66–1.73)
Income, NTD		
<250,000	426	1.00 (Reference)
250,000–299,999	515	1.21 (1.19–1.23)
300,000+	838	1.97 (1.94–2.00)
SO_2_, 5ppb increment		2.02 (2.00–2.04)
O_3_, 5ppb increment		0.98 (0.98–0.99)

RR, relative risk; CI, confidence interval.

## Data Availability

Data were retrieved from the insurance claims data of Taiwan (http://nhird.nhri.org.tw/, accessed on 9 October 2019) at the Health and Welfare Data Science Center, and access to this database can be requested by sending a formal proposal to the NHI.

## Supplementary Materials

The following supporting information can be downloaded at: https://www.mdpi.com/article/10.3390/v14091932/s1.

## Abbreviations

aRR	adjusted relative risk
CABs	childhood acute bronchitis and bronchiolitis
CI	confidence interval
cRR	crude relative risk
EPA	Environmental Protection Administration
ICD-9-CM/ICD-10-CM	International Classification of Diseases, 9th/10th Revisions, Clinical Modification
O_3_	ozone
PM_2.5_	particulate matter of ≤2.5 µm
RR	relative risk
RSV	respiratory syncytial virus
SO_2_	sulfur dioxide
