# Supplementary material for: Roles of Ambient Temperature and PM2.5 on Childhood Acute Bronchitis and Bronchiolitis from Viral Infection
Source: Viruses. 2022 Aug 30;14(9):1932. doi: 10.3390/v14091932 (PMC9503275; doi:10.3390/v14091932)

Supplement Figure S1. Lag 1-, 3- and 7-day adjusted relative risk (RR) and 95% confidence interval (CI) of acute bronchitis and acute bronchiolitis associated with temperature and $PM_{2.5}$ level controlling for income, SO_2 and O_3 levels

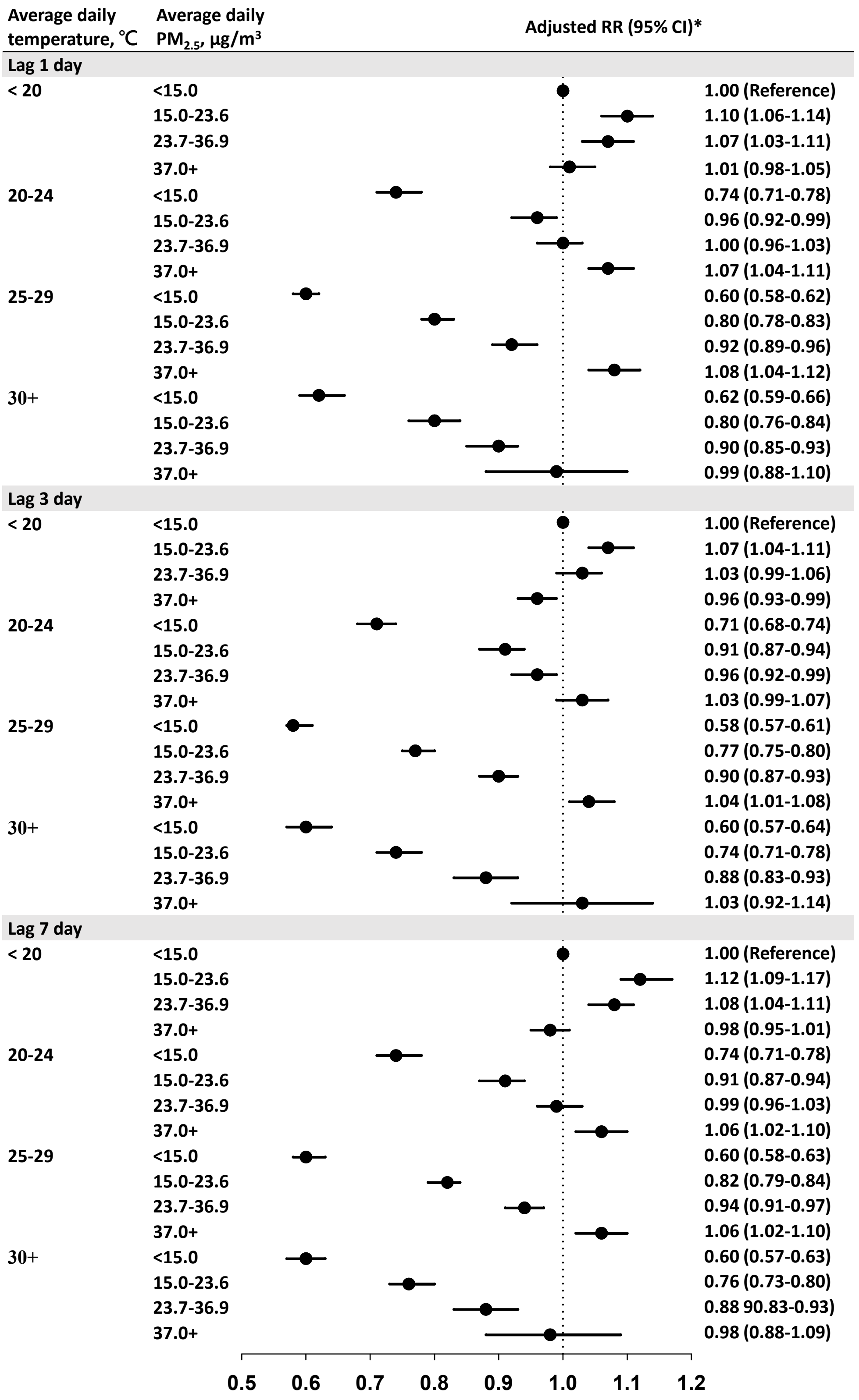

Supplement: Supplementary file 1 [file viruses-14-01932-s001.zip › viruses-1811972-supplementary.pdf]
